# Discovery of a Novel Hybrid of Vorinostat and Riluzole as a Potent Antitumor Agent

**DOI:** 10.3389/fcell.2020.00454

**Published:** 2020-07-14

**Authors:** Qifu Xu, Chunxi Liu, Jie Zang, Shuai Gao, C. James Chou, Yingjie Zhang

**Affiliations:** ^1^Department of Medicinal Chemistry, Key Laboratory of Chemical Biology (Ministry of Education), School of Pharmaceutical Sciences, Cheeloo College of Medicine, Shandong University, Jinan, China; ^2^Department of Pharmacy, Qilu Hospital, Cheeloo College of Medicine, Shandong University, Jinan, China; ^3^Department of Drug Discovery and Biomedical Sciences, South Carolina College of Pharmacy, Medical University of South Carolina, Charleston, SC, United States

**Keywords:** HDAC, inhibitor, drug design, hybrid molecule, anticancer

## Abstract

Vorinostat (suberoylanilide hydroxamic acid) was the first approved histone deacetylase (HDAC) inhibitor in a group of validated cancer therapeutic agents targeting epigenetics. Riluzole is a drug used to treat amyotrophic lateral sclerosis, the antitumor potency of which has been recently revealed. Herein, a novel hybrid of vorinostat and riluzole (compound **1**) was rationally designed, synthesized, and evaluated. Compared with vorinostat, compound **1** exhibited superior total HDAC inhibitory activity and similar HDAC isoform selective profiles. The intracellular HDAC inhibition of compound **1** was confirmed by Western blot analysis. Moreover, compound **1** possessed more potent *in vitro* antiproliferative activity against all tested solid and hematological tumor cell lines than vorinostat. *In vitro* metabolic stability evaluation of compound **1** revealed better human plasma stability and comparable human liver microsomal stability than vorinostat. Additionally, compound **1** demonstrated more significant *in vivo* antitumor activity in a MDA-MB-231 xenograft model than vorinostat, which could be attributed to its superior *in vitro* antiproliferative activity and metabolic stability. Taken together, the results presented here support further research and development of compound **1** as a promising antitumor agent.

## Introduction

**FIGURE 1:**
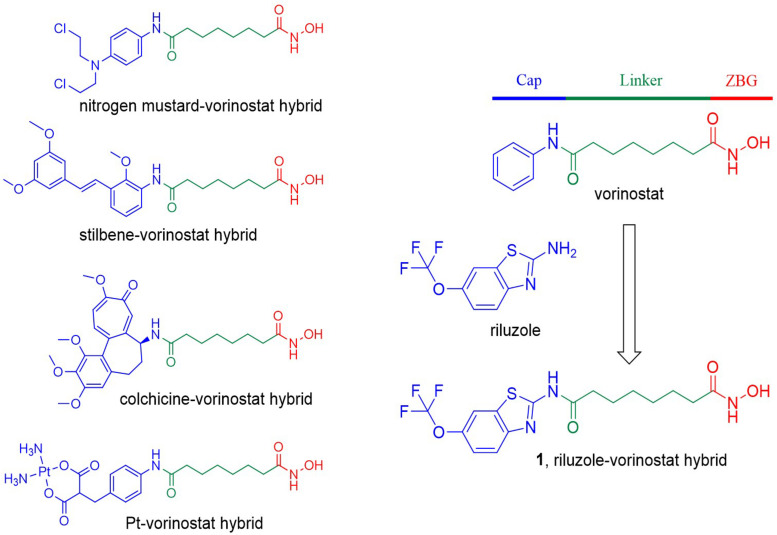
Compound design strategy by introducing various biologically active fragments to the terminal cap of vorinostat. The three parts of the HDAC inhibitor pharmacophore are indicated in different colors.

The acetylation status of lysine residues of nuclear histones, regulated by histone deacetylases (HDACs) and histone acetyl transferases (HATs), is one of the epigenetic mechanisms regulating gene expression (Allis and Jenuwein, 2016). Generally, HDAC overexpression causes a low histone acetylation level, which can downregulate the expression of many genes, including tumor suppressor genes, leading to cancer (Falkenberg and Johnstone, 2014). Therefore, targeting the HDAC family, especially the zinc-dependent HDACs, using small molecular inhibitors became a hot cancer therapeutic strategy, which has been well validated by the approval of five HDAC inhibitors for the treatment of hematological malignancies (Zagni et al., 2017). Vorinostat [suberoylanilide hydroxamic acid (SAHA); Figure 1] is the first approved HDAC inhibitor. Its structure summarizes well the common pharmacophore of most HDAC inhibitors, which contain a zinc binding group (ZBG) that chelates the catalytic zinc ion, a hydrophobic linker that occupies the tunnel of the active site, and a terminal cap that interacts with the amino acid residues around the entrance of the active site (Miller et al., 2003). Structural modification of the terminal cap of vorinostat is a feasible and efficient strategy to develop novel HDAC inhibitors. For example, the introduction of various biologically active fragments, including nitrogen mustard (Xie et al., 2017), proapoptotic stilbene (Giacomini et al., 2014), colchicine (Zhang et al., 2013), and platinum complex (Griffith et al., 2009), to the cap part of vorinostat successfully led to corresponding hybrid molecules with antitumor potency (Figure 1).

Riluzole is an approved drug for the treatment of amyotrophic lateral sclerosis (ALS; Bellingham, 2011). Many preclinical studies have revealed the anticancer potential of riluzole against breast cancer (Speyer et al., 2012, 2014, 2016; Teh et al., 2015), melanoma (Namkoong et al., 2007; Le et al., 2010; Khan et al., 2011; Lee et al., 2011; Wall et al., 2014; Wen et al., 2014; Rosenberg et al., 2015), glioma (Zhang et al., 2015), and prostate cancer (Akamatsu et al., 2009). Importantly, one preliminary clinical trial of riluzole in patients with resectable stage III or IV melanoma showed promising results (Yip et al., 2009). In addition, one clinical trial evaluating riluzole combined with sorafenib in patients with melanoma or advanced solid tumors is currently active (National Cancer Institute [NCI], 2011). It is worth noting that several analogs of vorinostat with benzothiazole cap groups were previously reported to show potent HDAC inhibitory and antitumor activity, suggesting that the introduction of benzothiazole-based riluzole to the terminal cap group of vorinostat can be tolerated (Tung et al., 2013). In the present study, because of its promising antitumor potency and appropriate physicochemical properties, riluzole was introduced to the terminal cap group of vorinostat, in the hope of obtaining a novel riluzole–vorinostat hybrid with potent HDAC inhibitory and antitumor activity (compound **1**; Figure 1).

## Materials and Methods

### Molecular Docking Study

Compound **1** was docked into the active site of HDAC2 (PDB code 4LXZ) using Tripos SYBYL-X 2.0. Before the docking process, the structure of the protein was treated by removing co-crystallized ligands, deleting water molecules, adding hydrogen atoms, and assigning AMBER7 FF99 charges. A 100-step energy minimization was performed to further optimize the protein structure. The molecular structure of compound **1** was generated with the Sybyl/Sketch module. It was optimized using Powell’s method with the TRIPOS force field with the convergence criterion set at 0.005 kcal/(Å mol) and assigned charges with the Gasteiger–Hückel method. Other parameters were set as default values. Molecular docking was carried out via the Sybyl/Surflex-Dock (SFXC) module.

### Chemistry

Unless specified otherwise, all starting materials, reagents, and solvents were commercially available. All reactions were monitored by thin-layer chromatography on 0.25 mm silica gel plates (60GF-254) and visualized with ultraviolet light, ferric chloride, or iodine vapor. Nuclear magnetic resonance (NMR) spectra were determined on Varian INOVA spectrometers, with δ in parts per million and *J* in Hertz, using tetramethylsilane as an internal standard. Measurements were made in dimethyl sulfoxide (DMSO)-*d*_6_ solutions. Electrospray ionization–mass spectrometry (ESI-MS) was carried out on an API 4000 spectrometer. High-resolution mass spectroscopy (HRMS) was conducted by the Shandong Analysis and Test Center. Silica gel was used for column chromatography purification. Melting points were determined on an electrothermal melting point apparatus and were uncorrected.

#### Procedure for the Synthesis of Octandioic Anhydride (3)

A solution of octandioic acid **2** (5.00 g, 28.7 mmol) in acetic anhydride (10 mL) was refluxed for 4 h. Then the mixture was dissolved in acetonitrile (60 mL) and frozen overnight. The resulting precipitate was filtered. The filter residue was dried to give compound **3** (2.78 g, yield 62%) as a light-yellow solid, which was used in the following reaction without further purification. ESI-MS *m/z*: 167.1 [M + H]^+^.

#### Procedure for the Synthesis of 8-oxo-8-((6-(Trifluoromethoxy)benzo[d]thiazol-2-yl) amino)octanoic Acid (4)

To a solution of compound **3** (1.17 g, 7.5 mmol) in tetrahydrofuran (THF; 50 mL) was added riluzole (1.17 g, 5.0 mmol). After reflux for 48 h, the solvent was removed under reduced pressure, followed by addition of ethyl acetate (EtOAc; 50 mL). The EtOAc solution was extracted with 1 M aqueous NaOH (3 × 20 mL). Then the aqueous phase was acidified until no precipitate appeared. The precipitate was filtered and the residue was dried to give compound **4** (1.56 g, yield 80%) as a white solid. ^1^H NMR (600 MHz, DMSO-*d*_6_) δ 12.45 (s, 1H), 12.00 (s, 1H), 8.11 (d, *J* = 1.8 Hz, 1H), 7.81 (d, *J* = 9.0 Hz, 1H), 7.42 (dd, *J* = 1.8 Hz, 9.0 Hz, 1H), 2.50–2.51 (m, 2H), 2.18–2.22 (m, 2H), 1.60–1.63 (m, 2H), 1.48–1.51 (m, 2H), 1.28–1.31 (m, 4H). HRMS [atmospheric pressure ESI (AP-ESI)] *m*/*z*: calculated for C_16_H_18_F_3_N_2_O_4_S [M + H]^+^ 391.0939; experimental 391.0924.

#### Procedure for the Synthesis of N1-Hydroxy-N8- (6-(Trifluoromethoxy)benzo[d]thiazol-2-yl) Octanediamide (1)

To a solution of compound **4** (0.78 g, 2.0 mmol) in THF (40 mL), triethylamine (Et_3_N) (0.22 g, 2.2 mmol) was added. Isobutyl chloroformate (0.30 g, 2.2 mmol) dissolved in THF (5 mL) was added to the reaction mixture in an ice bath, and the mixture was stirred for 1 h in an ice bath. A mixture of hydroxylamine hydrochloride (0.15 g, 2.2 mmol) and Et_3_N (0.22 g, 2.2 mmol) in methanol (5 mL) was stirred for 5 min then poured directly into the reaction mixture. The reaction continued for 4 h at room temperature, then the solvent was removed under reduced pressure followed by the addition of 30 mL of water. Then, 1 M HCl was used to adjust the pH to 6. The resulting precipitate was filtered and washed with water to obtain the crude product, which was purified by recrystallization to afford compound **1** (0.36 g, yield 45%) as a white solid. ^1^H NMR (600 MHz, DMSO-*d*_6_) δ 12.44 (s, 1H), 10.33 (s, 1H), 8.66 (s, 1H), 8.11 (d, *J* = 1.2 Hz, 1H), 7.80 (d, *J* = 9.0 Hz, 1H), 7.41 (dd, *J* = 1.2 Hz, 9.0 Hz, 1H), 2.50–2.51 (m, 2H), 1.93–1.95 (m, 2H), 1.59–1.64 (m, 2H), 1.47–1.51 (m, 2H), 1.26–1.30 (m, 4H). HRMS (AP-ESI) *m*/*z*: calculated for C_16_H_19_F_3_N_3_O_4_S [M + H]^+^ 406.1048; experimental 406.1055.

### Biology

#### *In vitro* HDAC Inhibition Fluorescent Assay

An aliquot of 10 μL of enzyme solution (HeLa cell nuclear extract, HDAC2, HDAC6, or HDAC8) was mixed with different concentrations of test compound (50 μL). The mixture was incubated at 37°C for 5 min, followed by the addition of 40 μL of fluorogenic substrate tert-butyl (S)-(6-acetamido- 1-((4-methyl-2-oxo-2H-chromen-7-yl)amino)-1-oxohexan-2-yl) carbamate (Boc-Lys(acetyl)-AMC) for HeLa cell nuclear extracts, HDAC2, and HDAC6; tert-butyl (S)-(1-((4-methyl- 2-oxo-2H-chromen-7-yl)amino)-1-oxo-6-(2,2,2-trifluoroacetam ido)hexan-2-yl)carbamate (Boc-Lys(trifluoroacetyl)-AMC) for HDAC8). After incubation at 37°C for 30 min, the mixture was quenched by the addition of 100 μL of developer containing trypsin and trichostatin A. Following incubation at 37°C for 20 min, the fluorescence intensity was measured using a microplate reader at excitation and emission wavelengths of 390 and 460 nm, respectively. The inhibition ratios were calculated from the fluorescence intensity readout of tested wells relative to those of control wells, and the half maximal inhibitory concentration (IC_50_) values were calculated using the prism non-linear curve-fitting method.

#### Western Blot Analysis

The MDA-MB-231 cells were treated with compounds or DMSO for a specified period of time. Then the cells were washed twice with cold phosphate-buffered saline (PBS) and lysed in ice-cold radioimmunoprecipitation assay (RIPA) buffer. Lysates were cleared by centrifugation. Protein concentrations were determined using the bicinchoninic acid assay. Equal amounts of cell extracts were then resolved by sodium dodecyl sulfate–polyacrylamide gel electrophoresis, transferred to nitrocellulose membranes, and probed with acetyl-histone H4 antibody (intracellular substrate of HDAC1 and HDAC2), acetyl-α-tubulin antibody (intracellular substrate of HDAC6), and β-actin antibody (used as a loading control), respectively. Blots were detected using an enhanced chemiluminescence system.

#### *In vitro* Antiproliferative Assay

All cell lines were maintained in Roswell Park Memorial Institute (RPMI) 1640 medium containing 10% fetal bovine serum at 37°C in a 5% CO_2_ humidified incubator. The cell proliferation assay was determined by the MTT (3-[4,5-dimethyl-2-thiazolyl]-2,5-diphenyl-2H-tetrazolium bromide) method. Briefly, cells were passaged the day before dosing into a 96-well plate, allowed to grow for 12 h, and then treated with different concentrations of compound for 72 h. A 0.5% MTT solution was added to each well. After incubation for another 4 h, formazan formed from MTT was extracted by adding 200 μL of DMSO. Absorbance was then determined using a microplate reader at 570 nm.

#### *In vitro* Metabolic Stability Assay in Human Plasma

Human plasma samples containing compound **1** were incubated at 37°C. At the specific time points, samples were added to acetonitrile to terminate the reaction, then subjected to vortex mixing for 5 min and stored in a freezer at –80°C. Before analysis, the samples were centrifuged. The remainder of **1** in the supernatants was analyzed by liquid chromatography with tandem mass spectrometry (LC-MS/MS). The *t*_1/2(half–time)_ values were calculated using the equation *t*_1/2(half–time)_ = −0.693/*k*, where *k* is the slope found in the linear fit of the natural logarithm of the fraction remaining of compound **1** versus incubation time.

#### *In vitro* Metabolic Stability Assay in Human Liver Microsomes

Human liver microsomes containing compound **1** were incubated with NADPH at 37°C. At the specific time points, samples were added to acetonitrile to terminate the reaction, then subjected to vortex mixing for 5 min and stored in a freezer at –80°C. Before analysis, the samples were centrifuged. The remainder of **1** in the supernatants was analyzed by LC-MS/MS. The *t*_1/2(half–time)_ values were calculated using the equation *t*_1/2_ = −0.693/*k*, where *k* is the slope found in the linear fit of the natural logarithm of the fraction remaining of compound **1** versus incubation time.

#### *In vivo* Antitumor Experiment Against MDA-MB-231 Xenograft

*In vivo* human tumor xenograft models were established as previously described (Zang et al., 2018; Liang et al., 2019). For the *in vivo* antitumor efficacy study, 5 × 10^6^ human breast cancer cells (MDA-MB-231) were inoculated subcutaneously in the right flank of female athymic nude mice (BALB/c-nu, 5–6 weeks old; Beijing HFK Bioscience Co., Ltd.). Ten days after injection, tumors were palpable and mice were randomized into treatment and control groups (six mice per group). The treatment groups received compound **1** or vorinostat by oral administration (30 mg/kg/day), and the blank control group received oral administration of an equal volume of PBS (5% DMSO). Subcutaneous tumors were measured with a vernier caliper every 3 days. Tumor volumes (*V*) were estimated using the equation (*V* = *ab*^2^/2, where *a* and *b* are the longest and shortest diameter, respectively). The body weight of the mice was also monitored regularly. At the end of the experimental period, mice were sacrificed and the tumor tissues were dissected and weighed. Tumor growth inhibition (TGI) and the relative increment ratio (T/C) were calculated at the end of treatment to reveal the antitumor effects in tumor weight and tumor volume, respectively.

TGI = (the mean tumor weight of control group – the mean tumor weight of treated group)/the mean tumor weight of control group.
T/C = the mean RTV of treated group (T)/the mean RTV of blank control group (C).

where the relative tumor volume (RTV) is *V*_*t*_/*V*_0_ (*V*_*t*_ is the tumor volume measured at the end of treatment; *V*_0_ is the tumor volume measured at the beginning of the treatment).

All the obtained data were used to evaluate the antitumor potency and toxicity of compounds. Data were analyzed by Student’s one-tailed *t-*test. A *p*-value of <0.05 was considered statistically significant.

## Results and Discussion

### Proposed Binding Mode of Compound 1 in HDAC2

**FIGURE 2:**
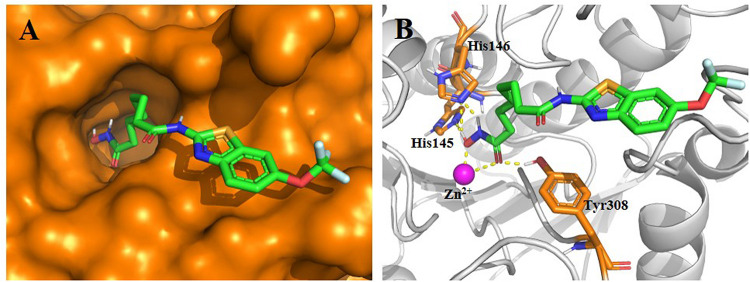
Proposed binding mode of compound **1** (green) with HDAC2 (PDB code 4LXZ). **(A)** Surface of HDAC2 with compound **1**. **(B)** Detailed interactions between HDAC2 and compound **1**. Yellow dashed lines represent the hydrogen bonds. Oxygen, nitrogen, fluorine, sulfur, and polar hydrogen atoms are shown in red, blue, pale cyan, bright orange, and white, respectively. The Zn^2+^ is shown as a magenta sphere. The figure was generated using PyMol (http://www.pymol.org/).

Before synthesis, a molecular docking study was performed to elucidate the potential binding mode of compound **1** in the active site of HDAC2. The results in Figure 2 indicate that compound **1** fitted well in the active site of HDAC2. In detail, the hydroxamic acid group of Compound **1** form four hydrogen bonds with His145, His146, and Tyr308, respectively. The aliphatic chain occupied the hydrophobic channel of the active site and the riluzole-based cap group occupied a shallow pocket around the entrance of the active site. The design strategy of compound **1** is rationalized via this binding mode.

### Synthesis

**SCHEME 1:**
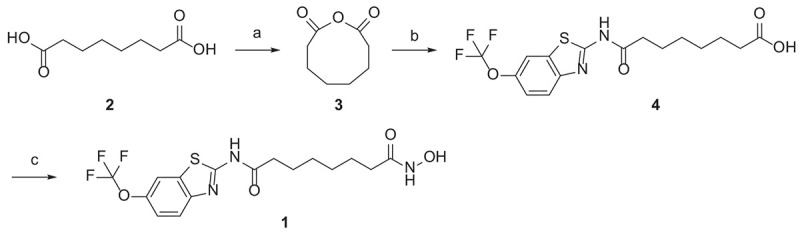
Reagents and conditions: **(a)** acetic anhydride, reflux; **(b)** riluzole, THF, reflux; **(c)** ClCOOi-Bu, TEA, NH2OH.HCl, THF, r.t.

The hybrid compound **1** was synthesized according to the procedures described in Scheme 1. The starting material, octandioic acid **2**, was refluxed in acetic anhydride to get the anhydride **3**, which reacted with riluzole to get the carboxylic acid **4**. Compound **4** was then condensed with hydroxylamine to get the target compound **1**.

### HDAC Inhibition and Isoform Selectivity

The total HDAC inhibitory activity of compound **1** was evaluated against the HeLa cell nuclear extract. The results listed in Table 1 show that compound **1** (IC_50_ = 0.12 μM) was more potent than the approved drug vorinostat (IC_50_ = 0.25 μM). To profile the HDAC isoform selectivity, compound **1** was further tested against HDAC2, HDAC6, and HDAC8 with vorinostat as the reference compound. The overall selectivity profile of compound **1** was similar to that of vorinostat (Table 1). Note that the HDAC6 inhibitory activity of **1** (IC_50_ = 0.012 μM) was more than sevenfold higher than that of vorinostat (IC_50_ = 0.091 μM).

**TABLE 1:** HDAC inhibition and isoform selectivity of compounds 1 and vorinostat.

**Compound**	**IC_50_ (μ M)^a^**			
	**HeLa nuclear extract**	**HDAC2**	**HDAC6**	**HDAC8**
**1**	0.12 ± 0.01	0.33 ± 0.04	0.012 ± 0.002	3.3 ± 0.2
Vorinostat	0.25 ± 0.02	0.23 ± 0.02	0.091 ± 0.004	>5

*^*a*^All assays were replicated (*n* = 3). The IC_50_ values are shown as mean ± SD.*

**FIGURE 3:**
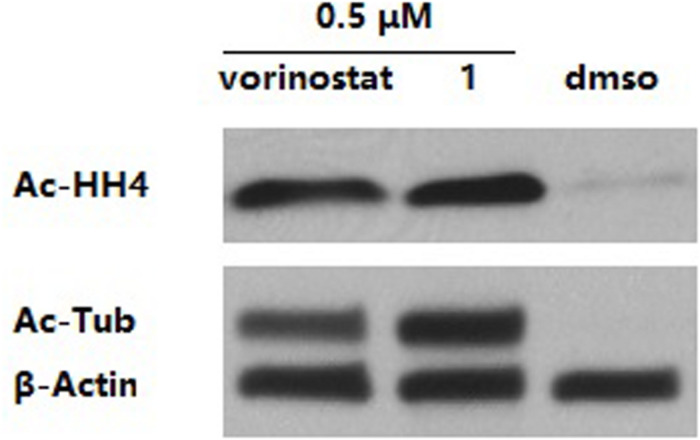
MDA-MB-231 cells were treated with dimethyl sulfoxide (DMSO) or compounds for 3 h. The levels of acetyl-α-tubulin (Ac-Tub) and acetyl-histone H4 (Ac-HH4) were determined by immunoblotting. β-Actin was used as a loading control. The result is a representative of three independent experiments.

Western blot analysis was performed to verify the intracellular target engagement of compound **1**. The results in Figure 3 show that both compound **1** and vorinostat could dramatically increase the levels of acetyl-histone H4 (intracellular substrate of HDAC1 and HDAC2) and acetyl-α-tubulin (intracellular substrate of HDAC6) in the MDA-MB-231 cell line. It is worth noting that, at the same concentration of 0.5 μM, the effect of **1** on acetyl-α-tubulin was superior to that of vorinostat, which is in line with the more potent HDAC6 inhibition of **1** compared with vorinostat, as shown in Table 1.

### *In vitro* Antiproliferative Activity

Because of its HDAC inhibitory potency, compound **1** was progressed to an *in vitro* antiproliferative assay against human tumor cells, including breast cancer cell lines MDA-MB-231 and MCF-7, prostate adenocarcinoma cell line PC-3, neuroblastoma cell line SK-N-BE(2), acute myelogenous leukemia cell line KG-1, acute lymphoblastic leukemia cell line MOLT-4, and erythroleukemia cell line HEL. Remarkably, compound **1** with IC_50_ values ranging from 0.14 to 2.74 μM was more potent against all tested human cancer cell lines than the approved drug vorinostat (Table 2). Note that riluzole showed less than 50% growth inhibition at 10 μM against the tested tumor cell lines, which was consistent with previous studies (Namkoong et al., 2007; Zhang et al., 2015; Speyer et al., 2016).

**TABLE 2:** *In vitro* antiproliferative activity of compound **1**, vorinostat, and riluzole.

**Compound**	**IC_50_ (μ M)^a^**						
	**MDA-MB-231**	**MCF-7**	**PC-3**	**SK-N-BE(2)**	**KG-1**	**MOLT-4**	**HEL**
**1**	0.77 ± 0.02	2.74 ± 0.06	2.52 ± 0.33	0.29 ± 0.007	0.65 ± 0.02	0.17 ± 0.007	0.14 ± 0.007
Vorinostat	1.58 ± 0.06	5.62 ± 0.06	9.21 ± 0.37	1.16 ± 0.27	1.59 ± 0.14	0.36 ± 0.049	0.21 ± 0.028
Riluzole	>10	ND^b^	>10	ND^b^	>10	ND^b^	>10

*^*a*^All assays were replicated (*n* = 3). The IC_50_ values are shown as mean ± SD. ^*b*^Not determined.*

### *In vitro* Metabolic Stability

One drawback of hydroxamate-based compounds, including vorinostat, is their poor metabolic stability. Therefore, the *in vitro* metabolic stability of compound **1** in human plasma and liver microsomes was assessed and compared with the reported data of vorinostat. The results in Table 3 show that, although compound **1** possessed similar stability in human liver microsomes to vorinostat (*t*_1/2_ = 56 min vs *t*_1/2_ = 60 min), its stability in human plasma was much better than that of vorinostat (*t*_1/2_ > 120 min vs *t*_1/2_ = 75 min).

**TABLE 3:** *In vitro* metabolic stability of compound **1** and vorinostat.

**Compound**	***t*_1/2_ (min)**	
	**Human plasma**	**Human liver microsomes**
**1**	>120^a^	56^a^
Vorinostat	75^b^	60^c^

*^*a*^All assays were replicated (*n* ≥ 2). ^*b*^Data from Konsoula and Jung (2008). ^*c*^Data from Venkatesh et al. (2007).*

### *In vivo* Antitumor Activity Assay

**FIGURE 4:**
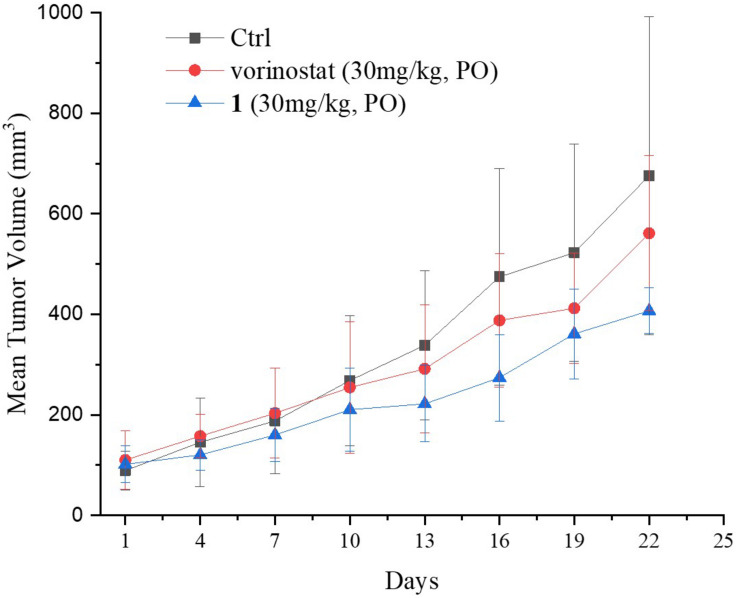
Growth curve of implanted MDA-MB-231 xenografts in nude mice. Data are expressed as the mean ± standard deviation.

**FIGURE 5:**
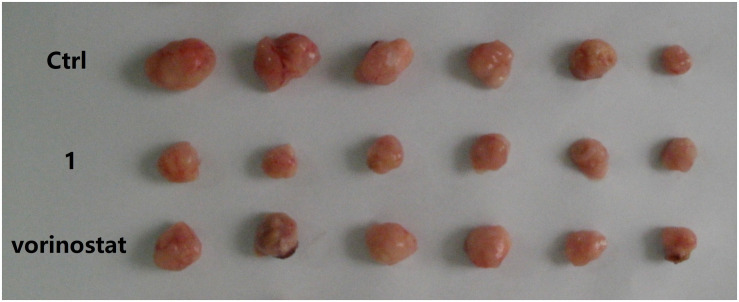
Dissected MDA-MB-231 tumor tissues.

**FIGURE 6:**
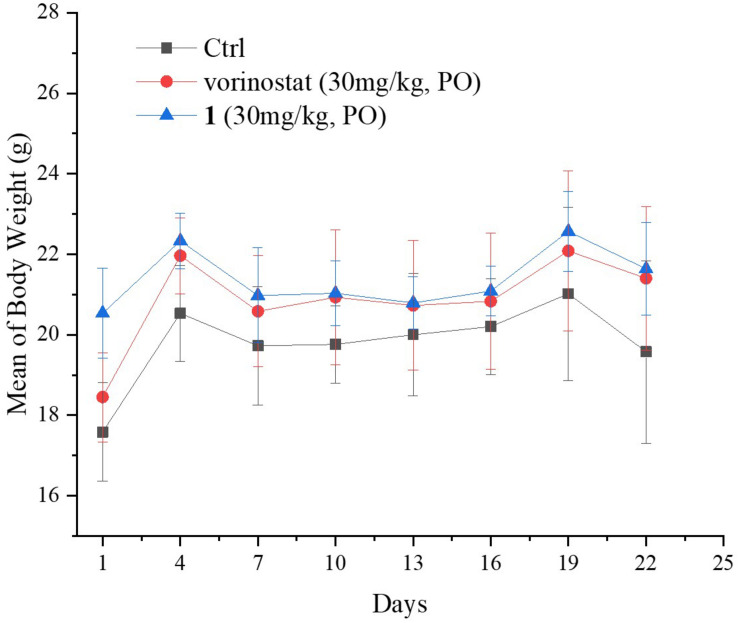
Animal body weight. Data are expressed as the mean ± standard deviation.

Based on the promising *in vitro* antiproliferative activity and metabolic stability of compound **1**, an MDA-MB-231 xenograft model was used to further evaluate the *in vivo* antitumor potency of compound **1**. After 21 consecutive days of treatment (30 mg/kg/day), TGI and T/C were calculated. As shown in Table 4, compound **1** demonstrated significantly better *in vivo* efficacy than vorinostat. The tumor growth curve and the final tumor tissue size are shown in Figures 4, 5, respectively, which explicitly demonstrate the potent antitumor activity of compound **1** *in vivo*. Moreover, the mouse body weights in Figure 6 indicate the high tolerability and low toxicity of compound **1**.

**TABLE 4:** *In vivo* antitumor activity in the MDA-MB-231 xenograft model^a^.

**Compound**	**TGI (%)**	**T/C (%)**
**1**	59	55
Vorinostat	33	78

*^*a*^Compared with the control group, compound **1** showed statistically significant (*p* < 0.05) tumor growth inhibition (TGI) and relative increment ratio (T/C) by Student’s one-tailed *t-*test, and vorinostat showed no statistically significant (*p* > 0.05) TGI and T/C by Student’s one-tailed *t-*test. The TGI values are based on tumor weight.*

## Conclusion

In the present work, based on the molecular hybridization strategy, a novel riluzole–vorinostat hybrid **1** was rationally designed, synthesized, and evaluated. Compared with vorinostat, compound **1** exhibited superior total HDAC inhibitory activity and similar HDAC isoform selective profiles, which was confirmed by Western blot analysis. Remarkably, compound **1** exhibited superior *in vitro* antitumor activity and metabolic stability to vorinostat, which contributed to its promising *in vivo* antitumor activity in the MDA-MB-231 xenograft model. In summary, our results support further mechanism studies and preclinical evaluation of compound **1** as a novel antitumor agent.

## Data Availability Statement

All datasets generated for this study are included in the article/supplementary material.

## Ethics Statement

The animal study was reviewed and approved by Shandong University Laboratory Animal Center Ethics Committee.

## Author Contributions

QX and CL contributed equally to research investigation, data analysis, manuscript writing, and organizing. JZ, CC, and SG performed the data validation and manuscript review. YZ contributed to conceptualization, supervision, and funding acquisition. All authors contributed to the article and approved the submitted version.

## Conflict of Interest

The authors declare that the research was conducted in the absence of any commercial or financial relationships that could be construed as a potential conflict of interest.
